# Ascending Aortic Surgery for Small Aneurysms in Men and Women

**DOI:** 10.21470/1678-9741-2022-0179

**Published:** 2023-10-23

**Authors:** Dmitri Panfilov, Victor Saushkin, Svetlana Sazonova, Boris Kozlov

**Affiliations:** 1 Cardiovascular Department, Cardiology Research Institute, Tomsk National Research Medical Center, Russian Academy of Sciences, Tomsk, Russian Federation; 2 Cardiac Imaging Department, Cardiology Research Institute, Tomsk National Research Medical Center, Russian Academy of Sciences, Tomsk, Russian Federation

**Keywords:** Aortic Aneurysm, Aortic Replacement, Sex Characteristics, Survival Analysis, Morbidity

## Abstract

**Introduction:**

According to recent data, thoracic aortic surgery has reduced morbidity and mortality including ascending aortic aneurysm treatment; however, women are at increased postoperative risk of adverse outcomes.

**Objective:**

Our aim was to evaluate early and late outcomes in male and female patients who underwent pre-emptive ascending aortic replacement (AAR).

**Methods:**

From January 2013 to September 2021, 91 patients (56 [61.5%] men and 35 [38.5%] women) underwent AAR for small (ranged from 5.0 to 5.5 cm) non-syndromic aneurysms. A propensity score-based adjustment of the groups was performed. We compared clinical outcomes between males and females.

**Results:**

Preoperative normalized aortic diameters were significantly larger in females (2.9 [2.7; 3.2] cm/m^2^) than in males (2.5 [2.3; 2.6] cm/m^2^, P<0.001), without differences in absolute values (51 [49; 53] mm vs. 52 [50; 53] mm, P=0.356). There were no significant differences in neurological, cardiac, pulmonary, and renal complications in both groups before and after matching. In-hospital mortality was 1 (1.8%) and 2 (5.7%) (P=0.307) in male and female patients in unmatched groups and 1 (2.9%) and 2 (5.7%) (P=0.553) in matched groups, respectively. Univariate logistic regression analysis revealed that the only risk factor for in-hospital mortality was age (odds ratio 1.117, 95% confidence interval 1.003-1.244; P=0.04). The overall survival rate was 83.5±0.06% in men and 94.3±0.04% in women at 36 months (P=0.404).

**Conclusion:**

Ascending aortic surgery for aneurysms ranged from 5.0 to 5.5 cm seems to have tolerable early and late outcomes in men and women.

## INTRODUCTION

**Table:** 

Abbreviations, Acronyms & Symbols	
AAR	= Ascending aortic replacement
AVR	= Aortic valve replacement
BMI	= Body mass index
BSA	= Body surface area
CABG	= Coronary artery bypass grafting
CI	= Confidence interval
CKD-EPI	= Chronic Kidney Disease Epidemiology Collaboration
ICU	= Intensive care unit
OR	= Odds ratio
SMD	= Standardized mean difference
TND	= Temporary neurological deficit

Ascending aortic aneurysm is a life-threatening condition with high rate of aortic-related deaths if left untreated^[1]^. Contemporary surgical management has reduced morbidity and mortality; however, the impact of sex-related differences is still poorly understood. Some authors found no differences in outcomes in both sexes^[2]^, but others show worse prognosis in women^[3-5]^. The explanation for inferior results in females is multifactorial and includes older age, smaller body size, and more comorbidities culminating in women having a lower physiological reserve for surgical procedures^[6,7]^.

It is known that early abdominal aortic repair at a lower size threshold for female patients are suggested to equalize outcomes between men and women^[7]^. Possibly, this strategy is also justified for women undergoing thoracic aortic surgery.

The purpose of our study was to analyze the early and late sex-related outcomes after ascending aortic surgery in patients with aneurysms ranged from 5.0 to 5.5 cm.

## METHODS

### Study Cohort

**Fig. 1:**
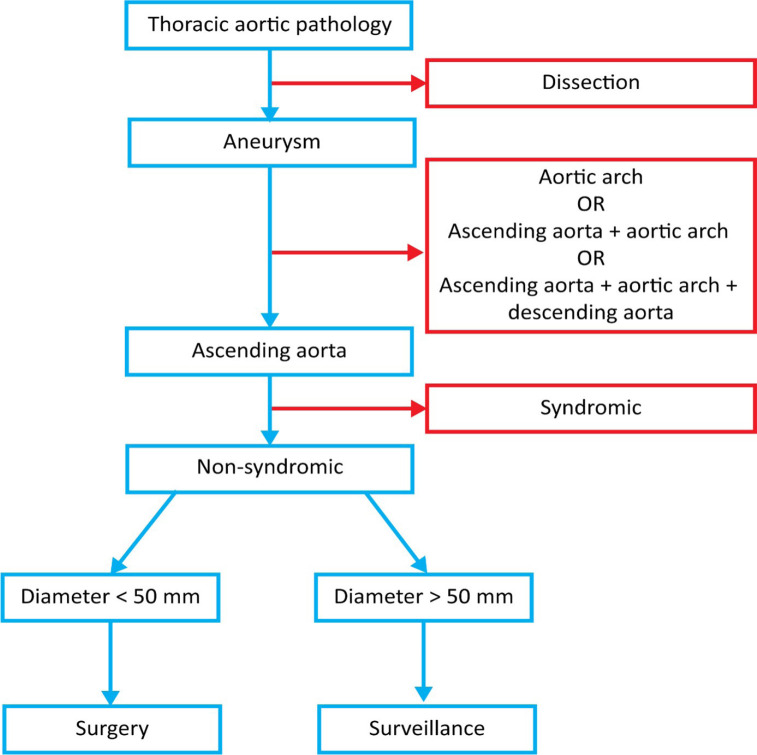
Flow diagram of the surgical strategy in patients with dilated ascending aorta.

**Fig. 2:**
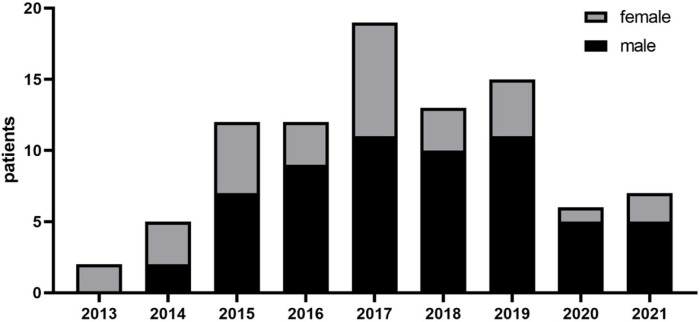
Distribution of surgical treatment by years in men and women.

Between January 2013 and December 2021, a total of 337 patients underwent thoracic aortic surgery at the Cardiology Research Institute, Tomsk National Research Medical Center. The retrospective study included 91 patients who underwent aortic surgery for non-syndromic asymptomatic ascending aortic aneurysms ranged from 5.0 to 5.5 cm (Figure 1). All patients were primarily stratified by sex into male and female groups (Figure 2). Patients with dissections or urgent/emergency cases or redo aortic surgery or requiring aortic arch surgery (total, subtotal, partial) were excluded from analysis. Baseline characteristics which included preoperative clinical status, details on surgery, postoperative outcome, and cause of death were compared between these groups. Follow-up data were prospectively recorded. The study was approved by the local Ethics Committee (#213), and all patients granted permission for the use of their medical records for research purposes.

### Analysis Endpoints

Primary endpoints were in-hospital mortality and follow-up death from any cause. Secondary endpoints were the incidence of neurological deficit (stroke and delirium), reoperation for bleeding, new-onset acute renal injury requiring renal replacement therapy (*i.e.*, haemodialysis or haemofiltration), and respiratory failure requiring prolonged lung ventilation (> 72 hours) or tracheostomy.

### Definitions

Small aneurysm was defined as a dilatation of the ascending aorta ranged from 5.0 cm to 5.5 cm. In-hospital mortality was defined as death within the same hospitalization of the index intervention. Postoperative stroke was defined as a neurological deficit that was confirmed postoperatively by means of computed tomography. Temporary neurological deficit was defined as a postoperative neurological deficit with a negative brain computed tomography scan and complete resolution at discharge.

### Surgical Technique

In all cases, the surgery was performed through a median sternotomy with cardiopulmonary bypass. Moderate-to-mild hypothermia (28-30°C) and antegrade cerebral perfusion via the innominate artery with an end-to-side graft were used for all patients. The distal aortic anastomosis was performed using open anastomosis fashion and involved resection of the inferior portion of the aortic arch from the base of the innominate artery to the projection of the origin of the left subclavian artery (hemiarch repair). Near infrared spectroscopy (INVOS™ 5100, Somanetics Corp., United States of America) was used for cerebral monitoring during the operation. When the target temperature was achieved, the arterial inflow rate was reduced to 800-1000 ml/min, and lower body circulatory arrest with antegrade cerebral perfusion was initiated. After opening of the aorta, distal aortic anastomosis was performed with a running 4/0 polypropylene suture with a Dacron® graft. When anastomosis was completed, rewarming of the patient was initiated. Proximal aortic anastomosis as well as simultaneous cardiac procedures (aortic valve replacement, coronary artery bypass grafting) were performed during the rewarming period. The patient was weaned from cardiopulmonary bypass when the body temperature reached 36°C.

### Aortic Imaging

All measurements were taken using electrocardiography-gated computed tomography angiography. Analysis was performed using 64-slice scanner Discovery NM-CT 570c (GE Healthcare, Milwaukee, Wisconsin, United States of America) with spatial resolution of the angiographic phase ranging from 0.6 to 1.25 mm. Adopted computed tomographic protocol included unenhanced, arterial, and delayed data acquisition. The arterial phase was acquired after intravenous injection of 80-100 mL of nonionic iodinated contrast at 5 mL/s, followed by a 50-mL bolus of saline solution. Delayed-phase scans were obtained 120-180 seconds after contrast injection. All measurements were taken in multiplanar reconstruction, always in the plane perpendicular to the manually corrected local aortic centre line. Ascending aortic diameter was measured at the level of the pulmonary artery bifurcation. The maximum aortic diameter (mm) was measured from the outer contours of the aortic wall. Normalized aortic diameter (cm/m^2^) was calculated by dividing the maximum aortic diameter (cm) by the body surface area (m^2^). The body surface area was calculated based on the Mosteller formula: body surface area (m^2^) = √ ([height (cm) × weight (kg)]/3600). Analysis and assessment of the images were based on the consensus between two experienced investigators.

### Follow-up

Follow-up was performed according to the institutional database supplemented by individual patient records. Clinical and radiologic follow-up was performed for all discharged patients (88 out of 91 operated patients [96.7%]). Mean follow-up time was 27±2.5 months (median, 17; range, 1-93). No patient was lost to follow-up. Data was obtained via records of clinical encounters or phone calls with patients and/or relatives. Postoperative computed tomography scans were performed for patients upon discharge, at six months from the last procedure, and annually thereafter. The number of postoperative computed tomography scans per patient was 3.9±2.4 (range, 0-10).

### Statistical Analysis

**Fig. 3:**
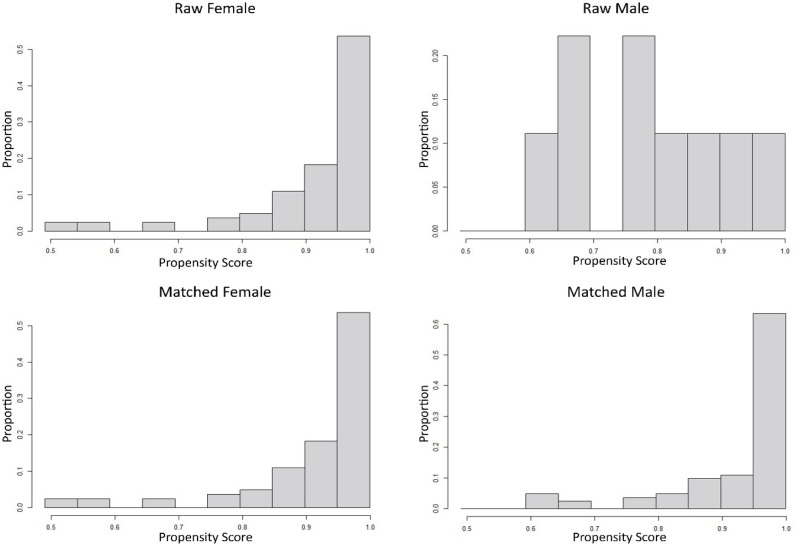
Mirror histogram before and after propensity score matching.

Categorical variables are summarized as n (%). Continuous data are described as median with the respective 25^th^ and 75^th^ percentiles. Normality was tested using the Shapiro-Wilk test. Baseline characteristics as well as intraoperative characteristics and postoperative outcomes are compared using the Mann-Whitney U test for continuous variables and the χ2 test for categorical variables (Fisher’s exact test was used when necessary due to the small cell sizes). Due to baseline differences between analyzed groups, we performed a propensity score-matched analysis matching variables and standardized mean difference. Variables included in propensity score-matched analysis were sex, age, body mass index, body surface area, aortic size, hypertension, atrial fibrillation, cerebral vascular accident, coronary artery disease, coronary artery bypass grafting, aortic valve replacement, temperature, operation time, cardiopulmonary bypass time, cardioplegic arrest time, and cerebral perfusion time. After calculating the nearest neighbor propensity scores, male patients were randomly sequenced and matched to female patients in 1:1 ratio using a caliper matching distance of 0.25 standard deviations of the logit of the estimated propensity score, resulting 35 pairs (Figure 3). Between-group comparisons were repeated in the propensity-matched population to ensure adequate balance in risk profile, and outcomes are then summarized in the matched population. Actuarial survival curves were estimated using the Kaplan-Meier method comparing differences between groups with log-rank test. Univariate logistic regression analysis was performed to identify risk factors of in-hospital mortality. Sex, age, body mass index, ascending aortic diameter, normalized aortic diameter, baseline comorbidities, ischemic times during surgery, and concomitant procedures were included in the analysis. *P*-value < 0.05 was used to define statistically significant difference. All analyses were performed using IBM Corp. Released 2013, IBM SPSS Statistics for Windows, version 22.0, Armonk, NY: IBM Corp., R version 3.3.1 (RStudio, United States of America), and Prism 8.2 (GraphPad Software, San Diego, California, United States of America).

## RESULTS

### Demographics and Clinical Presentation

Among 91 patients, 56 (61.5%) were men and 35 (38.5%) were women. The vast majority of patients’ preoperative characteristics had non-significant differences in both groups before matching (Table 1). The maximum ascending aortic diameter did not differ between females and males. Nevertheless, the normalized ascending aortic diameter had statistically significant difference (2.9 [2.7; 3.2] cm/m^2^ vs. 2.5 [2.3; 2.6] cm/m^2^, *P*<0.001) due to significantly lower body surface area in female patients (1.8 [1.6; 1.9] m^2^ *vs.* 2 [1.9; 2.2] m^2^, *P*<0.001). In addition, left ventricular ejection fraction was decreased in men compared to women (62 [54.5; 64] *vs.* 65.5 [63; 68], *P*<0.001). In contrast, preoperatively, women had slightly impaired renal function, when in men it was preserved (75.6±12.1 *vs.* 94.7±14.7, *P*<0.001). After matching males and females, creatinine level had a statistical difference. However, calculated glomerular filtration rate using the Chronic Kidney Disease Epidemiology Collaboration (or CKD-EPI) formula indicated that males and females were within one stage of chronic kidney disease (G2 - mildly decreased) with mean value of 79.2±2.6 and 77±3.1 ml/min/1.73 m^2^, respectively.

**Table 1:** Preoperative data.

Variable	Before matching				After matching			
	Men (n=56)	Women (n=35)	*P*-value	SMD	Men (n=35)	Women (n=35)	*P*-value	SMD
Age, years	60.5 [53.5; 65]	61 [54; 67]	0.526	0.247	60 [54; 63]	61 [54; 67]	0.869	0.065
> 70 years old, n (%)	5 (8.9%)	4 (11.4%)	0.697	0.077	4 (11.4%)	4 (11.4%)	> 0.999	0.001
BMI, kg/m^2^	28.9 [26; 32]	27.2 [24.4; 32.7]	0.225	0.174	29.4 [28; 31.5]	27.2 [24.4;32.7]	0.628	0.116
BSA, m^2^	2 [1.9; 2.2]	1.8 [1.6; 1.9]	< 0.001	-1.577	1.8 [1.8; 2]	1.8 [1.6; 1.9]	< 0.001	-0.876
Ascending aortic diameter, mm	51 [49; 53]	52 [50; 53]	0.356	0.061	51 [50; 52]	52 [50; 53]	0.508	0.155
Normalized aortic diameter, cm/m^2^	2.5 [2.3; 2.6]	2.9 [2.7; 3.2]	< 0.001	1.188	2.7 [2.5; 2.8]	2.9 [2.7; 3.2]	0.001	0.724
Hypertension, n (%)	38 (67.9%)	21 (60%)	0.445	-0.158	21 (60%)	21 (60%)	> 0.999	0.001
Diabetes mellitus, n (%)	5 (8.9%)	2 (5.7%)	0.575	-0.136	1 (2.9%)	2 (5.7%)	0.553	0.001
Coronary artery disease, n (%)	19 (33.9%)	8 (22.9%)	0.260	-0.260	10 (28.6%)	8 (22.9%)	0.456	0.001
Chronic obstructive pulmonary disease, n (%)	6 (10.7%)	1 (2.9%)	0.171	-0.465	1 (2.9%)	1 (2.9%)	> 0.999	0.001
Atrial fibrillation, n (%)	11 (19.6%)	3 (8.6%)	0.154	-0.390	4 (11.4%)	3 (8.6%)	0.553	0.001
Left ventricular ejection fraction, %	62 [54.5; 64]	65.5 [63; 68]	< 0.001	1.139	62.8 [56; 64]	65.5 [63; 68]	0.179	0.308
Left ventricular ejection fraction (< 40%), n (%)	3 (5.4%)	1 (2.9%)	0.571	-0.148	1 (2.9%)	1 (2.9%)	> 0.999	0.001
Creatinine, mg/dl	94.7±14.7	75.6±12.1	< 0.001	-1.544	85.1±13.2	75.6±12.1	< 0.001	-0.781
Glomerular filtration rate, ml/min/1.73 m^2^ (CKD-EPI formula)	79.2±2.6	77±3.1	0.627	-0.167	78.5±1.8	77±3.1	0.831	0.001
Previous cerebrovascular accident, n (%)	3 (5.4%)	5 (14.3%)	0.143	0.251	2 (5.4%)	5 (14.3%)	0.235	0.161
Previous cardiac surgery, n (%)	2 (3.6%)	1 (2.9%)	0.852	-0.042	1 (2.9%)	1 (2.9%)	> 0.999	0.001

BMI=body mass index; BSA=body surface area; CKD-EPI=Chronic Kidney Disease Epidemiology Collaboration; SMD=standardized mean difference

### Surgical Procedures

There were no differences in proximal aortic repair between males and females. All the patients predominantly underwent supracommissural aortic replacement. Aortic root replacement (Bentall or David procedure) was more frequent in men without significant differences. The frequency of the concomitant cardiac procedures did not differ in both groups. Operating, cardiopulmonary bypass, and cardioplegic arrest times were slightly longer in male patients, whereas duration of the cerebral perfusion with lower body circulatory arrest were identical in unmatched and matched groups (Table 2).

**Table 2:** Intraoperative data.

Variable	Before matching				After matching			
	Men (n=56)	Women (n=35)	*P*-value	SMD	Men (n=35)	Women (n=35)	*P*-value	SMD
Operation time, min	255 [220; 300]	240 [210; 330]	0.711	-0.016	272 [255;300]	240 [210;330]	0.812	-0.058
Cardiopulmonary bypass time, min	117 [99.5; 152.5]	115 [92; 145]	0.390	-0.153	125 [113;151]	115 [92;145]	0.908	-0.027
Cardioplegic arrest time, min	81.5 [72.5; 102.5]	78.5 [65; 115]	0.460	-0.020	81 [74; 100]	78.5 [65;115]	0.949	0.014
Lower body circulatory arrest, min	15 [14; 18]	15 [13; 18]	0.346	-0.227	15 [14;17]	15 [13;18]	0.596	-0.113
Cerebral perfusion time, min	15 [14; 18]	15.5 [14; 18]	0.993	-0.032	15.2 [15;18]	15.5 [14;18]	0.901	-0.027
Lowest temperature, °C	28 [26; 30]	27 [25; 29]	0.089	-0.390	27 [26;29]	27 [25;29]	0.431	-0.053
Supracommissural aortic replacement, n (%)	52 (92.8%)	33 (94.3%)	0.789	0.061	33 (94.3%)	33 (94.3%)	> 0.999	0.001
Bentall procedure, n (%)	1 (1.8%)	0	0.426	0.018	0	0	> 0.999	0.001
Aortic valve reimplantation (David technique), n (%)	3 (5.4%)	2 (5.7%)	0.942	0.015	2 (5.7%)	2 (5.7%)	> 0.999	0.001
CABG, n (%)	9 (16.1%)	4 (11.4%)	0.538	-0.144	5 (14.2%)	4 (11.4%)	0.553	0.001
AVR, n (%)	14 (25%)	9 (25.7%)	0.866	0.036	7 (20%)	9 (25.7%)	0.456	0.001

AVR=aortic valve replacement; CABG=coronary artery bypass grafting; SMD=standardized mean difference

### Outcomes

In-hospital outcomes are summarized in Table 3.

**Table 3:** Postoperative data.

Variable	Before matching				After matching			
	Men (n=56)	Women (n=35)	*P*-value	SMD	Men (n=35)	Women (n=35)	*P*-value	SMD
ICU stay, days	2 [2; 3]	2 [2; 6]	0.720	0.328	2 [2;3]	2 [2;6]	0.701	0.241
Invasive ventilation time, hours	13 [10; 20]	13.2 [10; 27]	0.542	0.362	13.1 [10; 20]	13.2 [10;27]	0.622	0.310
TND, n (%)	0	1 (2.9%)	0.203	0.169	0	1 (2.9%)	0.257	0.169
Stroke, n (%)	1 (1.8%)	0	0.426	-0.104	0	0	> 0.999	0.001
Delirium, n (%)	0	0	> 0.999	0.001	0	0	> 0.999	0.001
Respiratory failure, n (%)	2 (3.6%)	5 (14.3%)	0.059	0.302	2 (5.7%)	5 (14.3%)	0.235	0.241
Chest tube output, ml	300 [200; 400]	315 [200; 500]	0.605	0.143	308 [200; 400]	315 [200; 400]	0.723	0.078
Reoperation for bleeding, n (%)	0	2 (5.7%)	0.070	0.243	0	2 (5.7%)	0.154	0.243
Renal replacement therapy, n (%)	1 (1.8%)	2 (5.7%)	0.307	0.243	1 (2.9%)	2 (5.7%)	0.553	0.243
Myocardial infarction, n (%)	0	0	> 0.999	-0.142	0	0	> 0.999	-0.121
Mediastinitis, n (%)	1 (1.8%)	0	0.426	0.152	0	0	> 0.999	0.165
Multiple organ failure, n (%)	1 (1.8%)	1 (2.9%)	0.734	0.063	1 (2.9%)	1 (2.9%)	> 0.999	0.001
In-hospital mortality, n (%)	1 (1.8%)	2 (5.7%)	0.307	0.167	1 (2.9%)	2 (5.7%)	0.553	0.121

ICU=intensive care unit; SMD=standardized mean difference; TND=temporary neurological deficit

We observed no significant differences in temporary neurological deficit, stroke, or delirium between the analyzed groups. There were no cases of myocardial infarction in the cohort. The incidence of new-onset acute kidney injury and multiple organ failure was similar in women and men. At the same time, the rates of respiratory failure and reoperation for bleeding were higher in women but did not reach a significant difference.

**Fig. 4:**
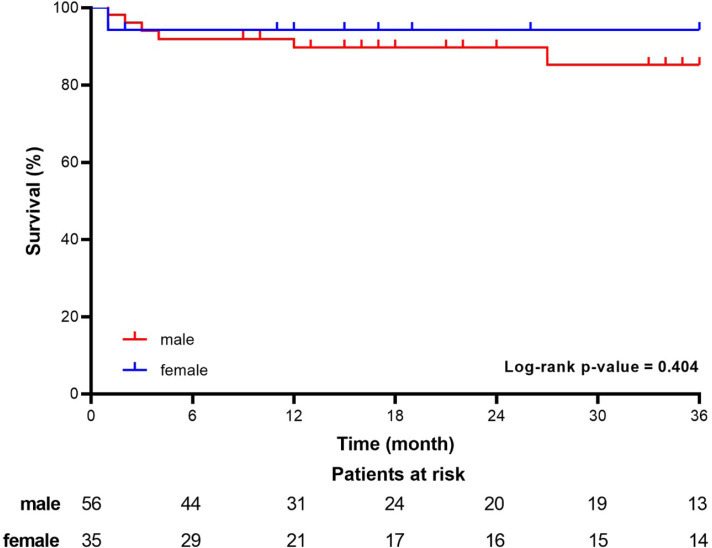
Kaplan-Meier survival curves for men and women after ascending aortic surgery.

In-hospital mortality did not differ between males and females and reached 1 (1.8%) and 2 (5.7%) (*P*=0.307) before matching and 1 (2.9%) and 2 (5.7%) (*P*=0.553) after matching, respectively. The causes of death were heart failure (n=1), multiple organ failure (n=1), and rupture of the aortic root (n=1). Late mortality was higher in men compared to women (5 [8.9%] *vs.* none, *P*=0.069). The causes of late death were rupture of the aortic root (n=1), rupture of the thoracoabdominal aorta (n=1), and Coronavirus disease 2019 (n=1). In two patients, cause of death was unknown. The overall survival rate at 36 months was 83.5±0.06% in male patients *vs.* 94.3±0.04% in female patients (*P*=0.404). No woman suffered from aortic-related events as rupture or dissection in the late follow-up period (Figure 4).

According to postoperative computed tomography scans the mean diameter of the replaced ascending aorta was 31±1.8 mm in men and 30.7±1.4 mm in women without significant differences between analyzed groups (*P*=0.843). During the follow-up, the aortic diameter was stable without increasing of the aortic size.

**Fig. 5:**
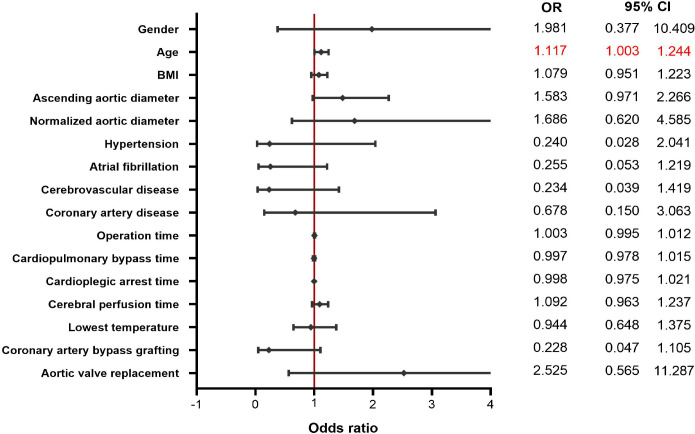
Sex-specific risk factors analysis for mortality in univariate logistic regression model. BMI=body mass index; CI=confidence interval; OR=odds ratio.

Univariate logistic regression analysis revealed that the only risk factor for in-hospital mortality was older age (odds ratio [OR] 1.117, 95% confidence interval [CI] 1.003-1.244; *P*=0.04). In a logistical model, female sex (OR 1.981, 95% CI 0.377-10.409, *P*=0.22), ascending aortic diameter (OR 1.483, 95% CI 0.971-2.266, *P*=0.07), as well as normalized aortic diameter (OR 1.68, 95% CI 0.620-4.585, *P*=0.27) did not influence significantly in-hospital mortality (Figure 5).

## DISCUSSION

Over the past decade, sex-related differences are gaining popularity in terms of incidence and outcomes in cardiovascular patients. Suboptimal results are described in females after coronary and valve surgery as well as for aneurysmal disease of the abdominal aorta^[8,9]^. However, it remains unclear whether this approach may benefit women undergoing thoracic aortic surgery^[7]^.

According to current guidelines, the indication for ascending aortic replacement is based on aortic diameter due to substantial impact of aortic size on aortic-related outcomes and death. The “borderline” for non-syndromic patients is ≥ 5.5 cm regardless of sex^[10,11]^. It is based on Elefteriades et al.^[12]^ study displaying a “hinge point” at 6 cm when the risk of rupture or dissection is increasing dramatically. Recently, they found a new hinge point at 5.25 cm, which calls to change the contemporary paradigm.

Apart from the aortic size, Davies et al.^[13]^ found that indexing aneurysm to body surface area is more important than absolute aortic size in predicting complications. Also, Matura et al.^[14]^ have shown that a normalized aortic diameter > 2.0 cm/m^2^ already presents an aortic enlargement, and diameters > 2.5 cm/m^2^ predispose to aortic dissection. Therefore, a high risk of adverse outcomes in large aneurysms of the ascending aorta motivated us to conduct the present study. We focused on sex-related differences after ascending aortic surgery in patients with small aneurysms.

In our study, mean aortic index in women was 2.9 cm/m^2^, and that was significantly higher compared to men. Considering this, females with the aortic size even below the cutoff value recommended in guidelines (5.5 cm)^[10,11]^ are still at high risk of aortic-related complications.

Aortic aneurysm growth rate may serve as a good reason for earlier aortic repair. Cheung et al.^[15]^ demonstrated aneurysm expansion as fast as three times in women than in men once aneurysm is indexed to body size. Additionally, Kim et al.^[16]^ identified female sex as a significant and independent risk factor of aortic expansion. For the purpose of explanation of the rapid growth rate in aneurysmal tissue in female patients, Sokolis et al.^[17]^ conducted their study. They found higher matrix metalloproteinases 2 and 9 and lower tissue inhibitors metalloproteinases in women than in men in ascending aortic aneurysms. Such breakdown of extracellular matrix with a significant deficiency in elastin and collagen mass in women promotes weakening of the aortic wall, resulting in increased risk of adverse events. Degeneration of the aortic wall increases due to decrease of the endogenous estrogen level in postmenopausal women^[18,19]^.

The other reason for pre-emptive aortic replacement in women is poorer outcomes. In Beller et al.^[20]^ study, 30-day mortality was 7.9% and 3.5% (*P*=0.058) for women and men, respectively. Similarly, Chung et al.^[3]^ have shown a significant sex difference in early mortality in a large cohort of patients (11% in women *vs.* 7.5% in men, *P*=0.02). We found no differences in early mortality between the analyzed groups. The in-hospital mortality rate in females and males was 5.7% and 1.8% (*P*=0.307), respectively. Our results are supported by another study. Friedrich et al.^[2]^ analyzed outcomes after ascending aortic repair in small aneurysms and did not reveal differences in early mortality between men and women. It is quite possible that large aortic size presented with fragile wall may hamper aortic reconstruction and may worsen surgical outcomes in women.

Additionally, we did not observe statistically significant sex differences in postoperative morbidity. The possible explanation for equal early results in our cohort may be similar baseline characteristics including age and comorbidity, ischemic times, concomitant procedures, and less complex proximal aortic reconstruction. Meanwhile, other studies documented an increased rate of postoperative neurological deficit, prolonged lung ventilation, and acute kidney injury in women^[2,20]^. In contrast, Friedrich et al.^[2]^ reported a high frequency of delirium and cerebrovascular accident as well as bronchopulmonary infection in male patients.

In our study, we defined age as the only risk factor for in-hospital mortality while female sex, aortic diameter, and aortic index did not reach statistical significance. Possibly, increasing sample size would exhibit these factors as statistically significant.

In the late follow-up, we recorded deaths in men only. Although we found no significant differences (*P*=0.404), the survival rate at three years was higher in women than in men (94.3±0.04% *vs.* 83.5±0.06%). At the same time, Beller et al.^[20]^ showed a significantly worse survival rate at four years in females than in males (84±3.6% *vs.* 92±1.7%, *P*=0.0052). Authors indicated that women were older with chronic obstructive pulmonary disease, hypertension, and more arch involvement that definitely influenced on outcomes.

Taking these data into account, the possibility of surgical intervention at a smaller ascending aortic size should be discussed. In accordance with the current aortic guidelines, aneurysms below the surgical thresholds size are subjected to conservative monitoring. At the same time, increased safety of surgery in high-volume aortic centers justifies a left-shift of the criterion for surgical aortic intervention^[21-23]^. Bearing this in mind, it seems logical to consider ascending aortic replacement for small aneurysms (in experienced centers) to improve outcomes, especially in females.

Summarizing our experience, findings of other authors^[7,23,24]^, and new epidemiological data^[25]^, it should be emphasized that there is a critical need to understand differences between males and females and to devise sex-specific strategies for ascending aortic aneurysm surveillance and treatment. Furthermore, there is a need to revise the current aortic guidelines with inclusion of sex-specific differences (aortic size thresholds, indexed values, etc.). Undoubtedly, it may be helpful to improve outcomes in both men and women.

### Limitations

This study has limitations, including the retrospective nature of a single-centre experience and small study population. So, there is a risk of a type II error due to a relatively small sample size. A relatively short follow-up period may not represent full picture of distant effects of the strategy of early aortic repair. However, this study is real-world based and reliable without patient selection bias. In addition, this study sheds light on this important field of evolving knowledge.

## CONCLUSION

Ascending aortic surgery for small aneurysms seems to have tolerable early and late outcomes. There were no differences in outcomes related to sex. Further investigations are required to determine a role of pre-emptive ascending aortic surgery in reducing the number of aortic-related events.
